# Genome Analysis of Alginate-Degrading Bacterium *Vibrio* sp. 32415 and Optimization of Alginate Lyase Production

**DOI:** 10.3390/microorganisms13102385

**Published:** 2025-10-16

**Authors:** Yi Zeng, Jia Xu, Zhongran Li, Rujie Wei, Haiyang Zhao, Liqin Sun, Chang Lu

**Affiliations:** Yantai Key Laboratory of Characteristic Agricultural Bioresource Conservation Germplasm Innovative Utilization, School of Life Sciences, Yantai University, Yantai 264000, China; 202300370048@s.ytu.edu.cn (Y.Z.); xj2417987186@163.com (J.X.); 19712021311@163.com (Z.L.); 19712026901@163.com (R.W.); zhaohaiyang616@outlook.com (H.Z.)

**Keywords:** *Vibrio*, alginate lyase, alginate-degrading bacterium, alginate metabolism

## Abstract

*Vibrio* sp. is one of the main producers of alginate lyase; however, most strains have problems such as low and unstable enzyme production. In this study, the enzyme production conditions of *V.* sp. 32415, a marine bacterium capable of producing extracellular alginate lyase, were optimized through Response Surface Design. The optimized medium was as follows: NaCl 12 g/L, FeSO_4_·7H_2_O 0.067 g/L, NH_4_Cl 7 g/L, alginate 11 g/L, K_2_HPO_4_·3H_2_O 4 g/L, MgSO_4_·7H_2_O 1 g/L. Under 28 °C, 160 rpm, 30 mL/300 mL liquid volume, and an initial pH 5.5 culture condition, the extracellular enzyme activity was 51.06 U/mL, which was 2.8 times higher compared with the activity before optimization. The optimal temperature, pH, and NaCl concentration for the extracellular alginate lyase were 37 °C, 8.0, and 0.1 M, respectively. The enzyme remained more than 80% of its original activity at 30 °C for 4 h. 1 mM Fe^3+^, Ca^2+^, K^+^, Mg^2+^, and Na^+^ enhance enzyme activity, with a preference for polyG blocks. *V.* sp. 32415 has two circular chromosomes and one circular plasmid. Chromosome 2 has two polysaccharide utilization loci. It utilizes alginate through the Scatter pathway. The results of this study provide theoretical and data support for understanding the production of extracellular alginate lyase by marine *Vibrio* and their metabolism and utilization of alginate.

## 1. Introduction

Alginate is an important component of the cell wall in brown algae. It is formed by the polymerization of β-D-mannuronic acid (M) and its isomer, α-L-guluronic acid (G), through (1,4) linkages, resulting in this type of linear polysaccharide. It typically accounts for 10–40% of the dry weight of the cell and plays a crucial role in maintaining the mechanical rigidity and flexibility of brown algae [1,2]. Due to its excellent gel-forming property, thickening ability and stability, alginate has been widely used in the food, chemical, textile and medical industries [3]. However, at the same time, its disadvantages such as large molecular weight and low water solubility have also limited its further application [4]. Compared with alginate, its degradation product, alginate oligosaccharides (AOS), not only retains the functions of polysaccharides, such as anti-tumor, growth promotion and immune regulation, but also has a simpler structure, shorter chain length, better water solubility and higher bioavailability [5].

Several methods can be employed to degrade alginate into AOS with a low degree of polymerization (DP), including physical, chemical and enzymatic degradation. Among all these methods, the chemical method is one of the fastest ways to produce AOS, but its reaction process is difficult to control, resulting in a random molecular weight distribution of the product [6]. In addition, it is less environmentally friendly. Physical methods can significantly reduce the degree of polymerization of alginate, but they usually consume a large amount of energy and incur high costs [7]. In contrast to physical and chemical methods, enzymatic degradation has the advantages of high efficiency, strong specificity, safety and minimal by-products [8].

The alginate lyase (EC 4.2.2.-) acts on the β-1, 4 glycosidic bonds through the β elimination mechanism, causing the breakdown of the alginate sugar chains and generating AOS [9]. Marine *Vibrio* sp. exhibits a wide range of metabolic profiles for organic carbon compounds and is one of the important producers of alginate lyase [10]. Currently, known strains capable of degrading and utilizing alginate include *V.* sp. W13 [11], *V. splendidus* OU02 [12], and *V. xiamenensis* QY104 [13], among others. The extracellular alginate lyase produced by them can be directly used for hydrolyzing brown algae [14] or preparing alginate oligosaccharides [15]. In addition, some alginate lyase genes, such as alyw208 [16], AlyC7 [17], AlgVR7 [18], have been isolated and characterized from the genomes of *Vibrio* sp.

Compared with other marine bacteria, the extracellular enzyme activity of marine *Vibrio* sp. is relatively low. For instance, the extracellular enzyme activity of *V.* sp. HB236076 is only 39.1 U/mL [15]. While the enzyme activity of *Paenibacillus algicola* HB172198T is 152 U/mL [19], and that of *Cobetia* sp. cqz5-12 is 160 U/mL [20]. Optimizing the composition of the culture medium or culture conditions is of great significance for enhancing the enzyme production in the strain. For example, Zhou et al. used Plackett–Burman Design and Central Composite Design to optimize the culture medium and conditions for *V*. sp. QY102, which produces alginate lyase [21]. After optimization, the yield of alginate lyase reached 52.8 U/mL, which was 329% higher than the control.

This study focused on an alginate-degrading bacterium strain *V*. sp. 32415 isolated from rotten kelp. Through whole-genome sequencing and analysis, the utilization sites and metabolic pathways of alginate were clarified. The enzyme production conditions were optimized using One-Factor-at-a-Time, Plackett–Burman Design, and Box–Behnken Design. The enzymatic properties of the extracellular enzymes were analyzed. The research results are beneficial for enriching the data of alginate lyase, understanding the metabolism and utilization of alginate by *Vibrio* sp., and providing data support and new resources for the biological enzymatic hydrolysis of AOS.

## 2. Materials and Methods

### 2.1. Screening and Identification of V. sp. 32415

One gram of rotten kelp was added to 100 mL of marine basal medium, using alginate as the carbon source, and the mixture was incubated at 28 °C and 120 rpm for 12 h. Single colonies were isolated through the selection of specific carbon sources of alginate and plate streaking. The plates were stained with 1 M CaCl_2_ solution. If a transparent circle appeared around the colony, this strain was identified as an alginate-degrading bacterium. Bacterial species were initially determined through sequencing and alignment of the 16S rRNA gene [20].

A Maximum Likelihood phylogenetic tree was constructed using MEGA 7.0.26 to determine the evolutionary position of *V*. sp. 32415. The sequence information used for tree construction is listed in Table S1. The tree-building model includes HKY+G+I, Gamma 2, and 1000 bootstrap replications. *Salinivibrio costicola* NCIMB 701^T^ was used as the outgroup [22].

The physiological and biochemical characteristics of the target strain *V*. sp. 32415 were analyzed and identified, including the range of growth temperature, pH and NaCl concentration, catalase, oxidase, ornithine decarboxylase, lysine decarboxylase, arginine decarboxylase, etc. The specific methods are referred to in the work of Kurilenko et al. [23].

### 2.2. Genome Sequencing and Annotation

*V*. sp. 32415 was expanded in Zobell 2216E medium, and the bacterial cells were collected by low-temperature centrifugation. The genomic sequencing and annotation were performed by Shanghai Biozeron Biotechnology Co., Ltd., Shanghai, China. The average nucleotide identity (ANI) and digital DNA-DNA hybridization (dDDH) were calculated using ANI calculator 1.0 (https://www.ezbiocloud.net/tools/ani, accessed on 24 May 2025) and Genome-to-Genome Distance Calculator 3.0 (http://ggdc.dsmz.de/ggdc.php, accessed on 23 May 2025), respectively [24,25].

### 2.3. Carbohydrate Utilization Capacity and Alginate Metabolism

CAZymes and carbohydrate-binding modules were predicted using NCBI CDD 1.0 (https://www.ncbi.nlm.nih.gov/Structure/cdd/wrpsb.cgi, accessed on 24 May 2025) and the CAZy database (http://www.cazy.org/, accessed on 24 May 2025), The metabolic pathway of alginate for *V*. sp. 32415 was constructed based on the Kyoto Encyclopedia of Genes and Genomes (KEGG) database and the dbCAN-PUL database https://pro.unl.edu/dbCAN_PUL/dbCAN_PUL/home, accessed on 24 May 2025).

The expression levels of alginate lyase genes in the strain were detected under different carbon sources using RT-qPCR [26]. gyrB was used as the internal reference gene.

### 2.4. Optimization of Enzyme Production

#### 2.4.1. Optimization of Medium Components

##### One-Factor-at-a-Time Experiment

Based on the marine basic medium (alginate 5 g/L, (NH_4_)_2_SO_4_ 5 g/L, K_2_HPO_4_·3H_2_O 2 g/L, NaCl 30 g/L, MgSO_4_·7H_2_O 1 g/L, FeSO_4_·7H_2_O 0.01 g/L, pH 7.5), the medium components were optimized through One-Factor-at-a-Time experiments. These factors included the carbon source (fucose, glucose, starch, sucrose, mannose, galactose, alginate), nitrogen source (NH_4_Cl, (NH_4_)_2_SO_4_, peptone, beef powder, urea, yeast extract), the concentration of the alginate (2 g/L–20 g/L), NH_4_Cl (2 g/L–20 g/L), NaCl (10 g/L–90 g/L), K_2_HPO_4_·3H_2_O (0 g/L–8 g/L), MgSO_4_·7H_2_O (0 g/L–6 g/L), FeSO_4_·7H_2_O (0 g/L–0.06 g/L), CaCl_2_ (0 g/L–0.05 g/L) and pH (4.0–9.0).

The strain was cultured at 28 °C and 120 rpm for 24 h. After the culture, the supernatant was collected by low-temperature centrifugation, and the alginate lyase activity in the supernatant was detected by the DNS method. One enzyme activity unit (U) was the amount of alginate lyase required to produce 1 μg of reduced sugar within 1 min. The optimal level of each single factor was determined based on this.

##### Plackett–Burman Experiment

The Design-Expert 13.0 software was used to design an experiment to determine the significant factors affecting the enzyme production of the strain. Seven factors, including alginate (A), NH_4_Cl (B), NaCl (C), K_2_HPO_4_·3H_2_O (D), MgSO_4_·7H_2_O (E), FeSO_4_·7H_2_O (F), and initial pH (G), were investigated. The optimal conditions obtained from the One-Factor-at-a-Time experiments were taken as the center values, and the high level (+1) and low level (−1) values were selected (Table S2). The enzyme activity of alginate lyase (Y) was used as the response value.

##### Box–Behnken Design

Taking the three significant factors obtained from the Plackett–Burman experiment as design factors and the enzyme activity value as the response value, the Box–Behnken design and response surface analysis were conducted using Design-Expert 13.0 to determine the optimal concentration of the main influencing factors and to carry out verification tests (Table S3).

#### 2.4.2. Optimization of Culture Conditions

A series of One-Factor-at-a-Time experiments was designed. Based on the optimal medium composition, the temperature (25–37 °C), rotation speed (80–180 rpm), and liquid volume (10 mL/300 mL–60 mL/300 mL) were changed. The enzymatic activity of alginate lyase in the fermentation supernatant was taken as the response value to analyze the effects of the above factors on the enzyme production of the strain.

### 2.5. Detection of Alginate Lyase Activity and Enzymatic Properties

The enzymatic properties of the extracellular alginate lyase from *V*. sp. 32415 were analyzed [27], including the effects of temperature, pH, NaCl concentration, metal ions on enzyme activity, thermal stability and pH stability of the enzyme, substrate preference, and enzyme kinetics analysis.

The enzymatic hydrolysis products of alginate, polyM and polyG by extracellular enzymes were analyzed by thin-layer chromatography. The developing solvent was n-butanol: acetic acid: water = 3:2:3 (*v*/*v*), and the staining reagent was ethanol: concentrated sulfuric acid = 9:1 (*v*/*v*). After staining, the samples were heated at 120 °C until the color developed [28].

## 3. Results and Discussion

### 3.1. Separation, Identification and Characterization of V. sp. 32415

**Figure 1:**
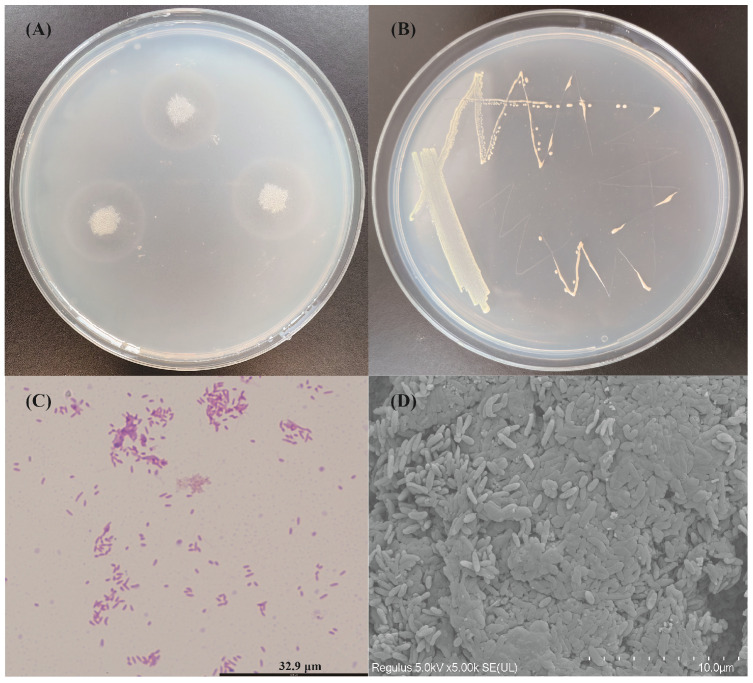
Identification of alginate lyase production capacity and morphological observation of *V*. sp. 32415. (**A**) The hydrolytic zone of *V*. sp. 32415 on marine basal medium plates containing 0.5% alginate as the sole carbon source. (**B**) Colony morphology. (**C**) Gram-staining (1000×). (**D**) Scanning electron microscopy image (10,000×).

After growing on marine basal medium plates containing 0.5% alginate at 30 °C for 48 h, *V*. sp. 32415 was treated with 1 M CaCl_2_ solution, and a transparent circle presented around the colonies (Figure 1A), indicating that 32415 could produce extracellular alginate lyase. On Zobell 2216E plates, *V*. sp. 32415 presented as neat-edged, milky white, circular colonies (Figure 1B). Its cells were Gram-negative (Figure 1C), aerobic, flagellum-free, non-motile, and curved, approximately 0.3–0.5 μm in width and 1.5–1.8 μm in length (Figure 1D).

The growth temperature of *V*. sp. 32415 ranges from 4 to 41 °C, pH from 4.0 to 9.0, and NaCl concentration from 5 to 90 g/L. The optimal growth temperature, pH and NaCl concentration are 20 °C, 7.5, and 50 g/L, respectively. It can utilize citrate, and is positive for urease, oxidase, catalase, arginine di-hydrolase, and nitrate reduction. It is negative for ornithine decarboxylase, lysine decarboxylase, arginine decarboxylase, tryptophan deaminase, and indole production [29].

**Figure 2:**
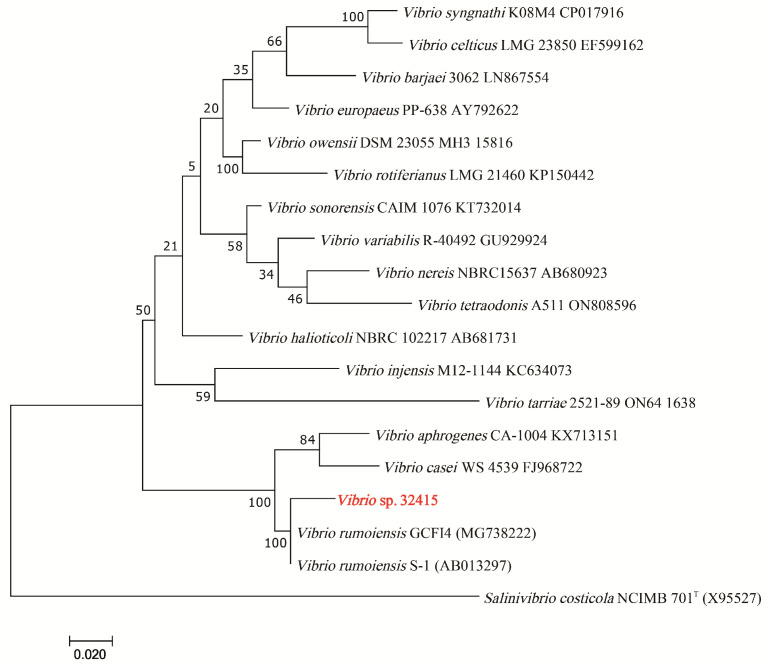
The Maximum Likelihood phylogenetic tree based on the 16S rRNA gene of *V.* sp., *Salinivibrio costicola* NCIMB 701^T^ is the outgroup, ^T^ means type strain.

The 16S rRNA gene of *V*. sp. 32415 (PX091797) was amplified and sequenced. The sequence was submitted to the NCBI database (https://blast.ncbi.nlm.nih.gov/Blast.cgi, accessed on 14 May 2025) for a BLAST search, and its similarity with *V. rumoiensis* PDI-3_MA (PP809126.1) and *V. algivorus* SA2 (AP018678.1) was 99.72% and 99.16%, respectively. A Maximum Likelihood phylogenetic tree was constructed based on the 16S rRNA gene sequence; it can be observed that *V*. sp. 32415 clustered with *V. rumoiensis* GCFI4 (MG738222) and *V. rumoiensis* S-1 (AB013297) (Figure 2).

To further clarify the taxonomic status of *V*. sp. 32415, dDDH (Table S4) and ANI (Table S5) were calculated based on its genomic data compared with other *Vibrio* species. The dDDH and ANI values between *V*. sp. 32415 and *V. rumoiensis* FERM P-14531 were the highest, reaching 79% and 98.64%, respectively. According to the species classification threshold, if dDDH > 70% and ANI > 95%, they can be considered as the same species [30]. Based on the above experimental results, *V*. sp. 32415 belongs to *V. rumoiensis*.

### 3.2. Genome Specifics

**Figure 3:**
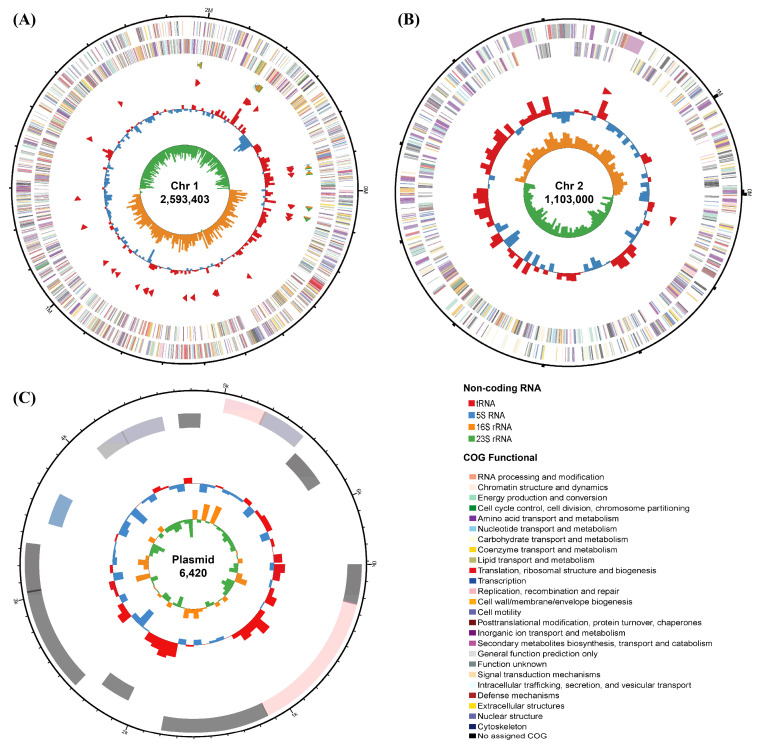
Genome map of *V*. sp. 32415. (**A**) Chromosome 1. (**B**) Chromosome 2. (**C**) Plasmid. The circles from the outer to the inner: Circle 1: the size of the genome. Circle 2 and 3: the CDS on the positive and negative strands, respectively, with different colors indicating the different COG functional classifications of the CDS. Circle 4: rRNA and tRNA. Circle 5: the GC content, with the outward red part indicating that the GC content of this region is higher than the average GC content of the whole genome. Circle 6: the GC skew value.

The genome of *V*. sp. 32415 is approximately 3.7 Mb, containing two circular chromosomes and one circular plasmid, with lengths of 2,593,403 bp, 1,103,000 bp, and 6420 bp for Chr1, Chr2, and plasmid, respectively (NCBI: PRJNA1302359) (Figure 3). A total of 3339 protein-coding sequences were predicted, with a total gene length of 3,210,126 bp, an average gene length of 961 bp, an average gene density of 0.901, and an average GC content of 42.6%. There are 93 tRNAs and 25 rRNAs. The N50 is 2,593,403 bp. These genomic features are consistent with the data of the known *Rumoiensis* clade [31].

### 3.3. Alginate Metabolism and Utilization

According to the CAZy database, the genome of *V*. sp. 32415 contains Auxiliary Activities (AA) 8, Carbohydrate-Binding Module (CBM) 18, Carbohydrate Esterase (CE) 11, Glycoside Hydrolase (GH) 32, Glycosyltransferase (GT) 28, and Polysaccharide Lyase (PL) 17. CAZymes account for 3.4% of the total protein-coding genes, and *V*. sp. 32415 can maintain its own growth by utilizing multiple carbon sources (Figure S1). Usually, the number of CAZymes in bacterial genomes does not exceed 2% [32]. In the alginate-degrading bacterium *V*. sp. HB236076, only 2.2% of the CAZymes were present [15]. These results indicate that *V*. sp. 32415 has a relatively good ability to utilize carbohydrates.

**Figure 4:**
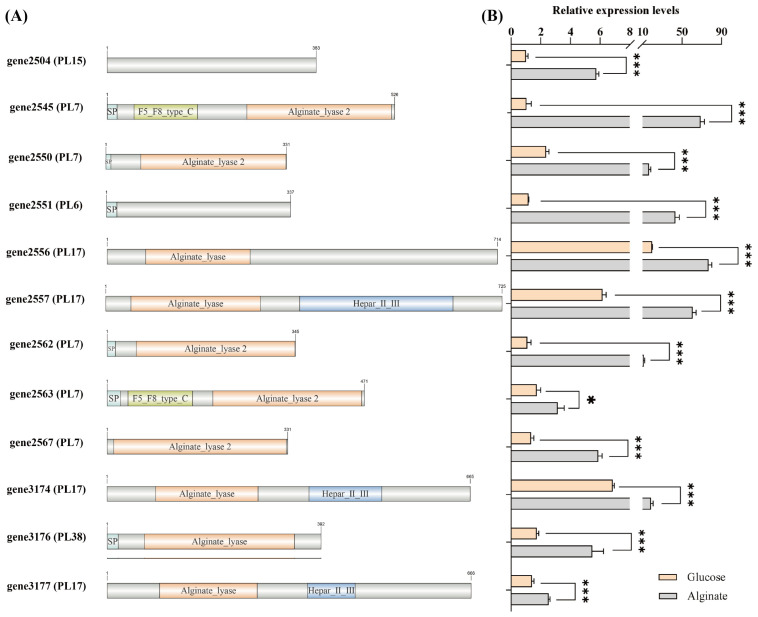
Analysis of the gene conserved domain and expression level of the alginate lyase in *V*. sp. 32415. (**A**) Conserved domain of alginate lyase. SP: Signal peptide. (**B**) Expression level of the alginate lyase gene after 24 h of cultivation. * *p* < 0.05, *** *p* < 0.001.

Among the 17 PL genes, a total of 12 alginate lyase genes were identified, which belong to different PL families (Figure 4A). This diverse composition of alginate lyase genes suggests that *V*. sp. 32415 may possess a complex and efficient enzyme system, with members within the system presumably having different enzymatic degradation patterns to cooperatively degrade high-molecular-weight substrates [33]. Although the sequences of gene2504 and gene2551 do not have the conserved domain of alginate lyase, they were still identified as alginate lyase and belong to the PL15 and PL6 families, respectively. The specific functions remain to be further verified.

The expression levels of alginate lyase genes proved that *V*. sp. 32415 belongs to the alginate-inducible enzyme-producing strain. During the culture process using alginate as the carbon source, the 12 predicted alginate lyase genes were significantly upregulated compared to glucose. Among them, the expression level of gene2545 was upregulated the most, by 66-fold compared to the control group, followed by gene2551 (37-fold) and gene2562 (10-fold) (Figure 4B).

**Figure 5:**
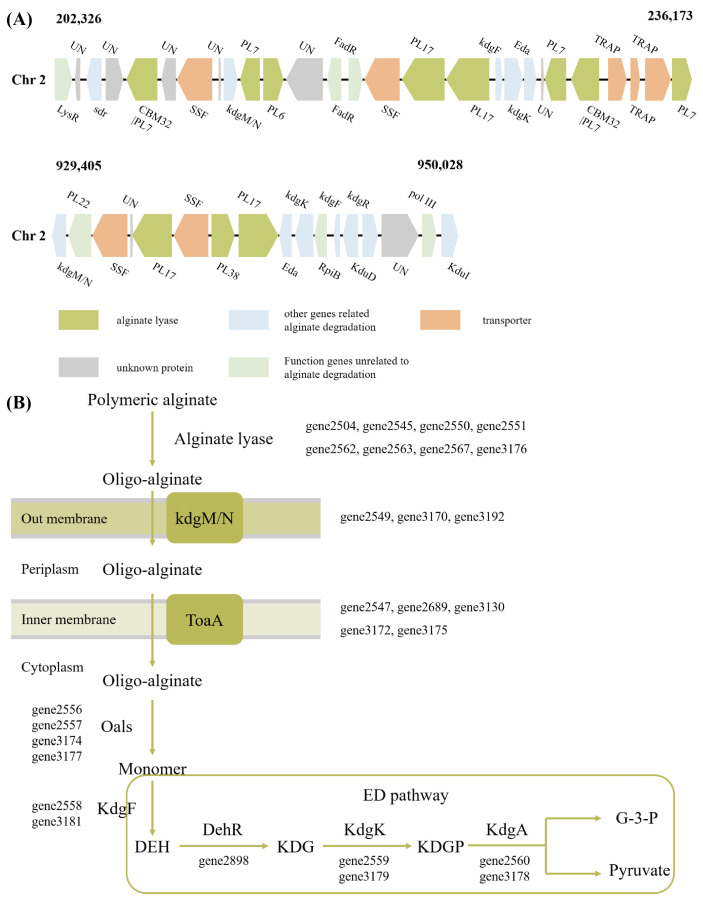
Degradation and utilization pathways of alginate in *V*. sp. 32415. (**A**) Alginate utilization loci of *V*. sp. 32415. (**B**) Alginate metabolic pathway of *V*. sp. 32415. LysR: transcriptional regulator, sdr: short-chain dehydrogenase/reductase, KdgR: transcriptional regulator KdgR, pol III: DNA polymerase III epsilon subunit, KduD: 2-deoxy-D-gluconate 3-dehydrogenase, KduI: predicted 4-deoxy-L-threo-5-hexosulose-uronate ketol-isomerase, KdgM/N: oligogalacturonate-specific porinin, ToaA/SSF: sodium-solute symporter, Oals: oligo-alginate lyases, KdgF: pectin degradation protein, DehR: 4-deoxy-L-erythrohexoseulose uronicacid reductase, kdgK: 2-dehydro-3-deoxygluconokinase, KdgA: 2-keto-3-deoxy-6-phospho-gluconate aldolase, DEH: 4-deoxy-L-erythro-hexoseulose uronic acid, KDG: 2-keto-3-deoxy-D-gluconate, KDPG: 2-keto-3-deoxy-6-phospho-gluconate, G-3-P: glyceraldehyde-3-phosphate.

Although Alginate Utilization Locus (AULs) are widely present in the genomes of *Vibrio* sp., most *Vibrio* sp. contain only one AUL. *V*. sp. 32415 has two AULs, both located on Chr2 (Figure 5A). The proteins contained therein are related to alginate degradation, transport and metabolism. Among the predicted 12 alginate lyase genes, 11 are located in AULs. Although gene2504 is also located on Chr2, due to its considerable distance (11.2 kb), it was not included in the two already discovered AULs. Multiple AULs may facilitate the faster and more active transcription of related genes in *V*. sp. in response to alginate metabolism [34].

*V*. sp. 32415 metabolizes and utilizes alginate through the Scatter system (Figure 5B). This pathway is common in *Vibrio* sp., characterized by the fact that genes related to alginate metabolism are not concentrated in a single operon but are scattered throughout the genome. Alginate is degraded extracellularly into AOSs, which are then transported into the cell via the kdgM/N and ToaA systems and further degraded into monosaccharides [35]. In contrast, for strains that utilize the PUL system to degrade alginate, such as *Microbulbifer* sp. HZ11 [26], the oligo-alginate lyases are usually located in the periplasm, where the degradation of oligosaccharides into monosaccharides takes place.

### 3.4. Optimization of Enzyme Production

#### 3.4.1. One-Factor-at-a-Time Fermentation Condition Optimization

**Figure 6:**
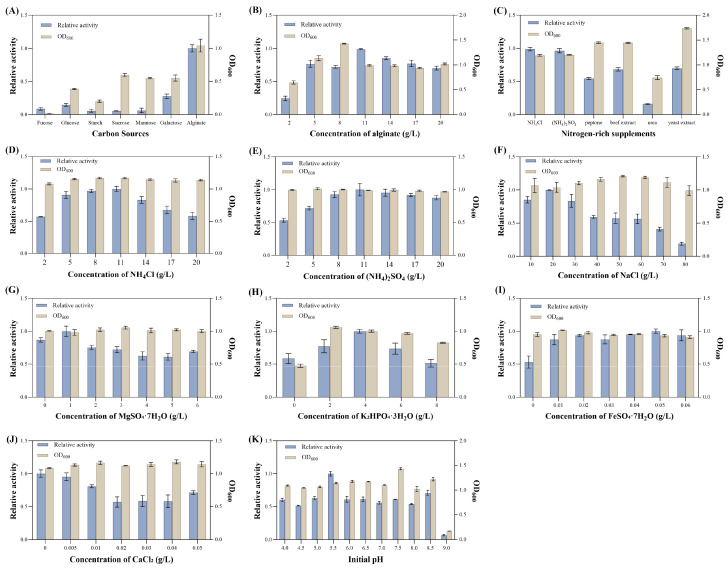
Effects of different factors on the growth and enzyme production of *V*. sp. 32415. (**A**) Carbon sources. (**B**) Alginate concentration. (**C**) Nitrogen-rich supplements. (**D**) NH_4_Cl concentration. (**E**) (NH_4_)_2_SO_4_ concentration. (**F**) NaCl concentration. (**G**) MgSO_4_·7H_2_O concentration. (**H**) K_2_HPO_4_·3H_2_O concentration. (**I**) FeSO_4_·7H_2_O concentration. (**J**) CaCl_2_ concentration. (**K**) Initial pH.

The effects of changes in medium components on the growth and alginate lyase activity of *V*. sp. 32415 were investigated. The results showed that when alginate was used as the carbon source, the extracellular alginate lyase activity of *V*. sp. 32415 was the highest, while for other carbon sources, the activity was almost zero, indicating that this strain requires a specific carbon source to induce enzyme production (Figure 6A). The relative enzyme activity was the highest when the alginate concentration was 11 g/L (Figure 6B). However, when the concentration exceeded this level, both the enzyme activity and the OD_600_ decreased, possibly due to the high viscosity of the alginate solution at high concentrations, which reduced the mass transfer rate of oxygen and led to insufficient oxygen supply, affecting the normal growth and enzyme production of *V*. sp. 32415 [36].

The optimal nitrogen-rich supplements for enzyme production were NH_4_Cl and (NH_4_)_2_SO_4_ (Figure 6C). Compared with the inorganic nitrogen-rich supplements, *V*. sp. 32415 produced less enzyme in the organic nitrogen-rich supplements, but the biomass of the strain increased significantly. While *Cobetia* sp. cqz5-12-M1 has a greater preference for organic nitrogen sources, the most suitable nitrogen source is yeast powder [37]. The total enzyme activity produced by *C*. sp. cqz5-12-M1 fermentation in organic nitrogen sources is higher than that in inorganic nitrogen sources. Urea had an inhibitory effect on the growth and enzyme production of *V*. sp. 32415. As the concentration of NH_4_Cl and (NH_4_)_2_SO_4_ increased, the extracellular enzyme activity was the highest within the 8–11 g/L, reaching 23.198 U/mL and 22.825 U/mL, respectively (Figure 6D,E). However, *V*. sp. 32415 seems to be more sensitive to high concentrations of NH_4_Cl, as the extracellular enzyme activity dropped more rapidly when the additive concentration exceeds 11 g/L.

Inorganic salts play an important role in maintaining the normal growth and metabolism of the strain. The strain could grow and produce enzymes within a range of 10 g/L to 80 g/L of NaCl concentration, indicating that the strain has a high tolerance to NaCl (Figure 6F). The optimal One-Factor-at-a-Time conditions for MgSO_4_·7H_2_O, K_2_HPO_4_·3H_2_O and FeSO_4_·7H_2_O concentrations were 1 g/L, 4 g/L and 0.05 g/L, respectively (Figure 6G–I). CaCl_2_ did not promote the enzyme production of *V*. sp. 32415. With the increase of CaCl_2_ concentration, the extracellular enzyme activity showed a downward trend. Therefore, the optimal addition amount of CaCl_2_ was 0 g/L (Figure 6J).

The relative enzyme activity was the highest when the initial pH was 5.5, and both strong acidic and strong alkaline conditions were unfavorable for bacterial growth and enzyme production (Figure 6K). Zhou et al. also found during the optimization of the enzyme-producing conditions of *V*. sp. QY102 that a weakly acidic initial pH (5.0) was beneficial for the enzyme production of the strain [21]. This was because during the fermentation process, alginate was degraded into oligosaccharides, resulting in an increase in the pH of the fermentation broth. A lower initial pH could play a certain role in controlling the pH changes in the fermentation broth. However, there are also some strains, such as *Enterobacter tabaci* RAU2C, that prefer an alkaline initial pH [38].

#### 3.4.2. Screening the Significant Factors Affecting the Enzyme Production of the Strain Through Plackett–Burman Experiment

In order to determine the crucial culture medium components that affect the enzyme production of *V*. sp. 32415, a group of Plackett–Burman Design including 12 experiment was designed using Design Expert software (version 13) to evaluate the seven variables and their levels listed in Table 1: A: alginate, B: NH_4_Cl, C: NaCl, D: K_2_HPO_4_·3H_2_O, E: MgSO_4_·7H_2_O, F: FeSO_4_·7H_2_O, G: pH.

In the 12 experiments, the alginate lyase activity (Y) varied within the range of 13.348 U/mL to 36.055 U/mL (Table 1). The total model *p* value was 0.009, the F value was 15.82, indicating that this model was significant and the results were reliable. The determination coefficient R^2^ was 0.9651, indicating that 96% of the variation in alginate lyase production could be explained by this model. The adjusted determination coefficient R^2^_adj_ is a modification of the ordinary determination coefficient R^2^. The higher the R^2^_adj_, the more concise and effective the model is in explaining the data. In this experiment, R^2^_adj_ was 0.9042, indicating that 90.42% of the data fluctuations can be explained by the model. The precision is the ratio of the effective signal to noise (Adequate Precision). In this experiment, the precision was 11.8741, which was much higher than the reasonable threshold of 4.0, indicating that the results of the Plackett–Burman Design can be used for the subsequent analysis of the Box–Behnken design [39,40].

The *p* values of NaCl, FeSO_4_·7H_2_O, and NH_4_Cl concentrations were all less than 0.05, suggesting that these three factors had significant effects on the enzyme production of the strain and should be further optimized (Table S6). Among them, NaCl and NH_4_Cl concentrations had negative effects, while FeSO_4_·7H_2_O concentration had a positive effect. However, Alginate, K_2_HPO_4_·3H_2_O, MgSO_4_·7H_2_O, and pH concentration had no significant impact on enzyme activity (Figure S2). Through multiple regression fitting of the data, the regression equation was obtained:

Y = 27.55 + 1.38A − 1.86B − 5.9C − 0.098D + 1.38E + 2.34F + 0.1388G(1)

#### 3.4.3. Box–Behnken Design Optimization Factor Parameters

Based on the Plackett–Burman experiment, to further determine the levels of key factors affecting enzyme production, a 3-level 3-factor Box–Behnken experiment was designed using the Design Expert software (version 13). Its results are shown in Table 2 and Table S7. The overall model *p* value < 0.01, indicating that the model was highly significant. The *p* value of the lack-of-fit term was 0.9291, which was greater than 0.05, suggesting that the lack-of-fit term was not significant. The above two results demonstrated the reliability of the model. The multiple determination coefficient R^2^ of the equation was 0.9709, and the adjusted coefficient R^2^_adj_ was 0.9335, indicating that the regression equation had a good fit and could well explain the changes in the enzyme activity of *V*. sp. 32415. From the F value, it can be seen that the influence on enzyme activity is A (NaCl) > C (NH_4_Cl) > B (FeSO_4_·7H_2_O).

**Figure 7:**
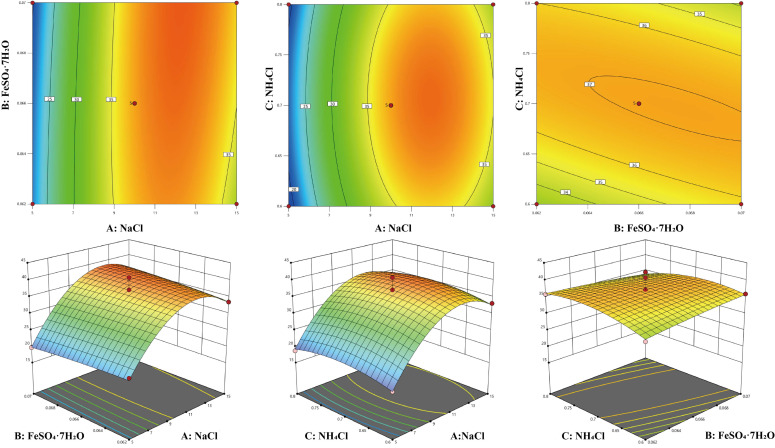
Three-dimensional diagram and contour map of the response surface experiment. The color changes from blue to red, indicating that the response value (extracellular enzyme activity) varies from small to large.

Contour plots and three-dimensional response surface plots can be used to describe the relationship between variables and response values. Using Design-Expert 13.0 software, the response surface analysis plots of AB, AC, and BC in the regression equation were drawn (Figure 7). In the response surface plots, a convex surface indicates that there is a maximum point between two factors, which also indicates that there is a maximum enzyme activity point in the experiment. In the contour plots, an elliptical shape indicates a significant interaction between two factors. From the amplitude of the response surface curve changes, it can be seen that the steepness of the NaCl concentration is the greatest, indicating that it has the most significant effect on the enzyme production of the strain, followed by NH_4_Cl and FeSO_4_·7H_2_O. This result is consistent with the analysis in Table S7.

#### 3.4.4. Verification of Optimization Results

According to the regression model, the optimal combination for the Box–Behnken experiment was determined to be NaCl 12 g/L, FeSO_4_·7H_2_O 0.067 g/L, and NH_4_Cl 7 g/L. The remaining values were alginate 11 g/L, K_2_HPO_4_·3H_2_O 4 g/L, MgSO_4_·7H_2_O 1 g/L. The initial pH was 5.5. The predicted optimal enzyme activity value was 38.886 U/mL. Three replicate experiments were conducted using this optimal combination to verify the results. The average enzyme activity value was 37.579 U/mL, which was close to the predicted optimal enzyme activity value, indicating that the optimized regression equation is reliable.

#### 3.4.5. Fermentation Condition Optimization

**Figure 8:**
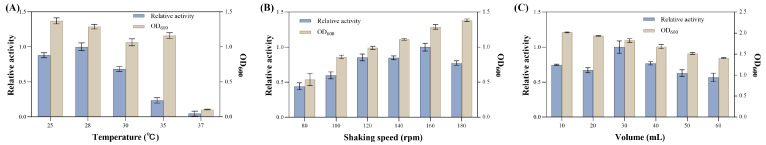
Effects of fermentation conditions on strain growth and enzyme production. (**A**) Temperature. (**B**) Shaking speed. (**C**) Liquid loading volume.

At a temperature of 28 °C, the extracellular enzyme activity is the highest. When the temperature exceeds 30 °C, the enzyme activity rapidly decreases. At 37 °C, the strain hardly grows and the extracellular enzyme activity approaches zero (Figure 8A). Therefore, the temperature of 28 °C is selected for the subsequent experiments. The extracellular enzyme activity of the strain reaches its peak at 160 rpm, and further increasing the rotation speed leads to a decrease in enzyme activity. However, within the experimental range, the biomass of the strain continues to increase with the increasing rotation speed (Figure 8B). The rotation speed affects the oxygen transfer rate and cell shear stress, thereby influencing the growth and enzyme production of the strain [41]. Considering all these factors, the rotation speed of 160 rpm is selected for the subsequent experiments. The effect of the liquid loading volume on enzyme production was studied. When the liquid loading volume is 30 mL/300 mL, the enzyme activity is the highest, and the other liquid loading volumes have little effect on the enzyme activity (Figure 8C).

#### 3.4.6. Growth and Enzyme Production Curves Before and After Optimization

**Figure 9:**
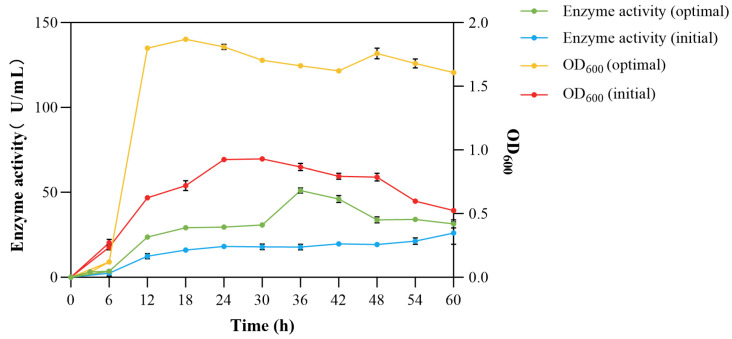
Growth and enzyme production curves of *V*. sp. 32415 before and after optimization.

After optimizing the components of the culture medium and fermentation conditions, the enzyme activity and strain biomass clearly increased. It began to enter the stable growth phase after 12 h, 12 h earlier than the original conditions. In terms of enzyme production, the optimized enzyme activity reached the highest of 51.062 U/mL, which was 2.8 times that of the original 18.176 U/mL (Figure 9). The shortening of the fermentation time reduced the risk of contamination by miscellaneous bacteria, decreased the probability of potential economic losses, and also reduced the time cost. It has potential application value in industrialization [42].

### 3.5. Enzymatic Properties of Extracellular Alginate Lyase

**Figure 10:**
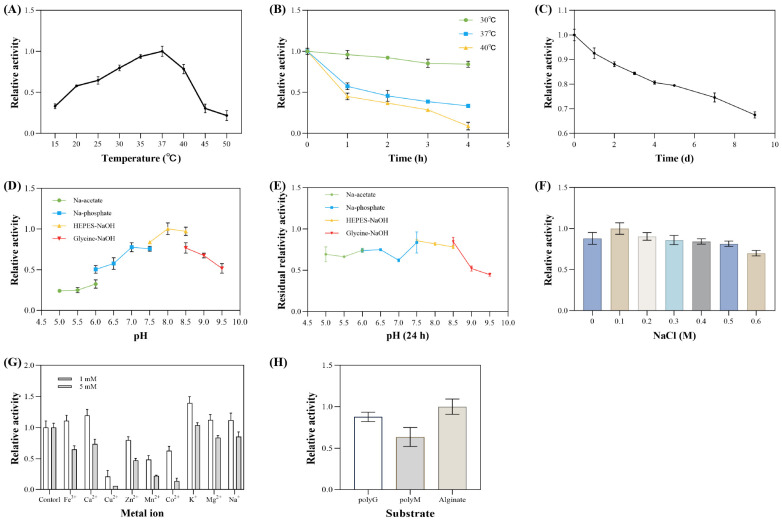
Enzymatic properties of *V*. sp. 32415 extracellular alginate lyase. (**A**) Temperature. (**B**) Thermal stability. (**C**) Low-temperature stability. (**D**) pH. (**E**) pH stability. (**F**) NaCl concentration. (**G**) Metal ions. (**H**) Substrate specificity.

The enzymatic properties of the extracellular alginate lyase from *V*. sp. 32415 were analyzed and characterized. The extracellular enzyme exhibited the highest activity at 37 °C. Within the temperature range of 20–40 °C, the enzyme activity was at least 50% of its maximum value. When the temperature exceeded 45 °C, the enzyme activity decreased rapidly (Figure 10A). Since most alginate lyases are derived from marine microorganisms, their optimal temperatures are mostly concentrated in the range of 30–40 °C [43,44]. However, some alginate lyases show cold adaptation or heat resistance. For example, the optimal temperature of alyRm3 from *Rhodothermus marinus* is 75 °C, and after incubation at 70 °C for 16 h, the remaining activity was 40% [45]. In contrast, the optimal temperature of TAPL7B was 20 °C, and the activity decreased rapidly when the temperature exceeded 35 °C [46].

In the experiment on the thermal stability of extracellular enzymes, after incubation at 30 °C and 37 °C for 4 h, the enzyme activity remained at 84.2% and 33.5% of the original activity, respectively. After incubation at 40 °C for 3 h, the enzyme activity decreased significantly, retaining only 28.6% of the original activity. After 4 h of incubation, the enzyme activity was almost completely lost (Figure 10B). After incubation at 4 °C for 9 days, approximately 66% of the initial enzyme activity was retained (Figure 10C).

The optimal pH of this extracellular enzyme is 8.0. When the pH is lower than 7, the enzyme activity drops rapidly (Figure 10D). It remains relatively stable at pH levels ranging from 5.0 to 8.5. After incubation for 24 h at acidic and neutral pH values, the enzyme activity remains above 60% (Figure 10E).

The optimal NaCl concentration was 0.1 M (Figure 10F). To adapt to the high-salt environment of the ocean, most alginate lyases show NaCl dependence or salt activation [16]. Compared with high salt concentrations, this extracellular enzyme exhibits greater enzymatic activity at low salt concentrations. However, this enzyme is not sensitive to changes in NaCl concentration, retaining 88% and 70% of the maximum enzymatic activity at 0 M and 0.6 M NaCl, respectively. This indicates that the extracellular enzyme is a salt-tolerant enzyme and can play a role in the degradation of substrates under low and high salt conditions [47].

When 1 mM of metal ions were added to the enzyme reaction system, Fe^3+^, Ca^2+^, K^+^, Mg^2+^, and Na^+^ all showed varying degrees of promoting effects on enzyme activity. Among these, K^+^ had the best promoting effect, being 1.39 times that of the control group. When 5 mM of metal ions were added to the system, except for K^+^ which had no significant effect, the other metal ions all showed inhibitory effects on enzyme activity. Cu^2+^ had a strong inhibitory effect on the enzyme, resulting in almost zero enzyme activity. The inhibitory effect of Mn^2+^ was also significant, with enzyme activity only 21% of the control group (Figure 10G). Metal ions may affect enzyme activity by interacting with the active center of the enzyme or the substrate binding site, influencing the conformation of enzyme or substrate binding capacity [48].

Interestingly, the optimal conditions for enzyme production by *V*. sp. 32415 do not fully align with the optimal reaction conditions of its extracellular alginate lyase. For instance, the optimal temperature for the extracellular enzyme is 37 °C (Figure 10A), but the strain hardly grows at this temperature (Figure 8A). The extracellular enzyme prefers a weakly alkaline environment (Figure 10D), yet the strain has the highest enzyme production capacity at an initial pH of 5.5 (Figure 6K).

When *V*. sp. 32415 fermented using alginate as the carbon source, the pH of the fermentation broth increases with the extension of fermentation time. It is possible that the metabolic products or the products generated by extracellular enzymatic hydrolysis during the strain’s fermentation process affect the pH of the fermentation broth [21]. It led to multiple variables in fermentation processes. This is a limitation of shake flask batch culture that cannot be ignored. Using continuous culture (such as small-scale chemostats) might more effectively control variables and more accurately reflect the influence of different factors on bacterial growth and enzyme production, helping to clarify the underlying regulatory mechanisms.

**Figure 11:**
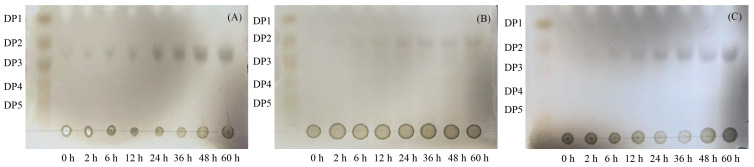
Degradation products of *V*. sp. 32415 were analyzed by TLC. (**A**) Alginate. (**B**) polyM. (**C**) polyG.

The interior of a microbial cell is like a precisely operating “factory”, which needs to find a balance condition to enable multiple catalytic enzymes to function normally and meet the growth and metabolic needs of the cell. Additionally, the thermal stability of enzymes must also be considered. Although the optimal temperature for the extracellular alginate lyase produced by *V*. sp. 32415 was 37 °C, it remained about 50% of its initial activity after incubation at 37 °C for 1 h; its stability at 30 °C was better than at 37 °C (Figure 10C). Similar “deviations” have also been observed in other studies [41,49]. Therefore, we speculate that this difference reflects the parameter changes during the fermentation process and the biological trade-offs between environmental adaptation and growth metabolism in microorganisms. The enzymatic hydrolysate of this enzyme was analyzed by TLC (Figure 11). After 2 h of enzymatic hydrolysis, AOS could be detected. The degradation products of alginate, polyM and polyG were mainly disaccharides.

## 4. Conclusions

The effects of cultivation conditions and medium components on the enzyme production of *V*. sp. 32415 were investigated. The results of the Plackett–Burman experiment indicated that the concentrations of NaCl, NH_4_Cl, and FeSO_4_·7H_2_O were significant influencing factors for enzyme production. The optimized medium composition was: NaCl 12 g/L, FeSO_4_·7H_2_O 0.067 g/L, NH_4_Cl 7 g/L, alginate 11 g/L, K_2_HPO_4_·3H_2_O 4 g/L, and MgSO_4_·7H_2_O 1 g/L. When cultivated under the conditions of 28 °C, 160 rpm, 30 mL/300 mL liquid volume, and an initial pH 5.5, the extracellular enzyme activity reached 51.062 U/mL, which was 2.8 times higher than before optimization. Through response surface optimization, the enzyme production of the strain was effectively improved. The genome of *V*. sp. 32415 contains two AULs. The extracellular alginate lyase degrades high-molecular-weight alginate into disaccharides, and the strain further metabolizes them through the Scatter pathway. These research results are helpful for a deeper understanding of the production of alginate lyase and the utilization of alginate in *Vibrio* sp.

## Figures and Tables

**Table 1:** Plackett–Burman experiment design and response values.

Number	Level							Enzyme Activity (U/mL)
	Alginate (g/L)	NH_4_Cl (g/L)	NaCl (g/L)	K_2_HPO_4_·3H_2_O (g/L)	MgSO_4_·7H_2_O (g/L)	FeSO_4_·7H_2_O (g/L)	G: pH	
1	14	14	10	2	0	0.06	5	36.055
2	14	8	30	6	2	0.04	5	25.295
3	14	8	10	2	2	0.04	6	34.879
4	14	14	10	6	2	0.06	5	34.275
5	14	14	30	2	0	0.04	6	17.828
6	14	8	30	6	0	0.06	6	25.233
7	8	8	10	2	0	0.04	5	28.921
8	8	8	30	2	2	0.06	5	26.543
9	8	14	10	6	2	0.04	6	30.941
10	8	8	10	6	0	0.06	6	35.579
11	8	14	30	2	2	0.06	6	21.643
12	8	14	30	6	0	0.04	5	13.348

**Table 2:** Design and results of Box–Behnken experiment.

Number	Level			Enzyme Activity (U/mL)
	NaCl (g/L)	FeSO_4_·7H_2_O (g/L)	NH_4_Cl (g/L)	
1	20	0.05	11	40.704
2	10	0.06	11	19.687
3	30	0.04	11	33.554
4	20	0.05	11	37.142
5	20	0.06	8	35.935
6	10	0.04	11	22.441
7	20	0.04	14	35.848
8	30	0.05	14	33.304
9	20	0.04	8	32.322
10	20	0.05	11	34.439
11	20	0.06	14	34.459
12	20	0.05	11	36.178
13	10	0.05	14	18.686
14	20	0.05	11	36.816
15	30	0.05	8	33.109
16	10	0.05	8	18.636
17	30	0.06	11	35.418

## Data Availability

The data presented in this study are openly available in NCBI, https://www.ncbi.nlm.nih.gov/genbank/ accessed on 1 July 2025.

## Supplementary Materials

The following supporting information can be downloaded at: https://www.mdpi.com/article/10.3390/microorganisms13102385/s1, Figure S1: Growth status of *V*. sp. 32415 using different carbon sources; Figure S2: Pareto chart showing the significance of factors affecting alginate lyase production by *V*. sp. 32415 based on Plackett–Burman Design; Table S1: 16S rRNA sequences from all the selected species for phylogenetic analysis; Table S2: Plackett–Burman experimental design and results; Table S3: Response surface experimental design; Table S4: dDDH values between *Vibrio* sp. 32415 and other *Vibrio* genus strain; Table S5: ANI values between *Vibrio* sp. 32415 and other *Vibrio* genus strain; Table S6: Plackett–Burman test design analysis; Table S7: Analysis of variance of response surface model.

## Abbreviations

The following abbreviations are used in this manuscript:

AOS	Alginate oligosaccharides
AUL	Alginate Utilization Locus
polyM	Poly mannuronic acid
polyG	Poly guluronic acid
PUL	Polysaccharide Utilization Locus
