# Supplementary figures and images for: Genome Analysis of Alginate-Degrading Bacterium Vibrio sp. 32415 and Optimization of Alginate Lyase Production
Source: Microorganisms. 2025 Oct 16;13(10):2385. doi: 10.3390/microorganisms13102385 (PMC12565957; doi:10.3390/microorganisms13102385)

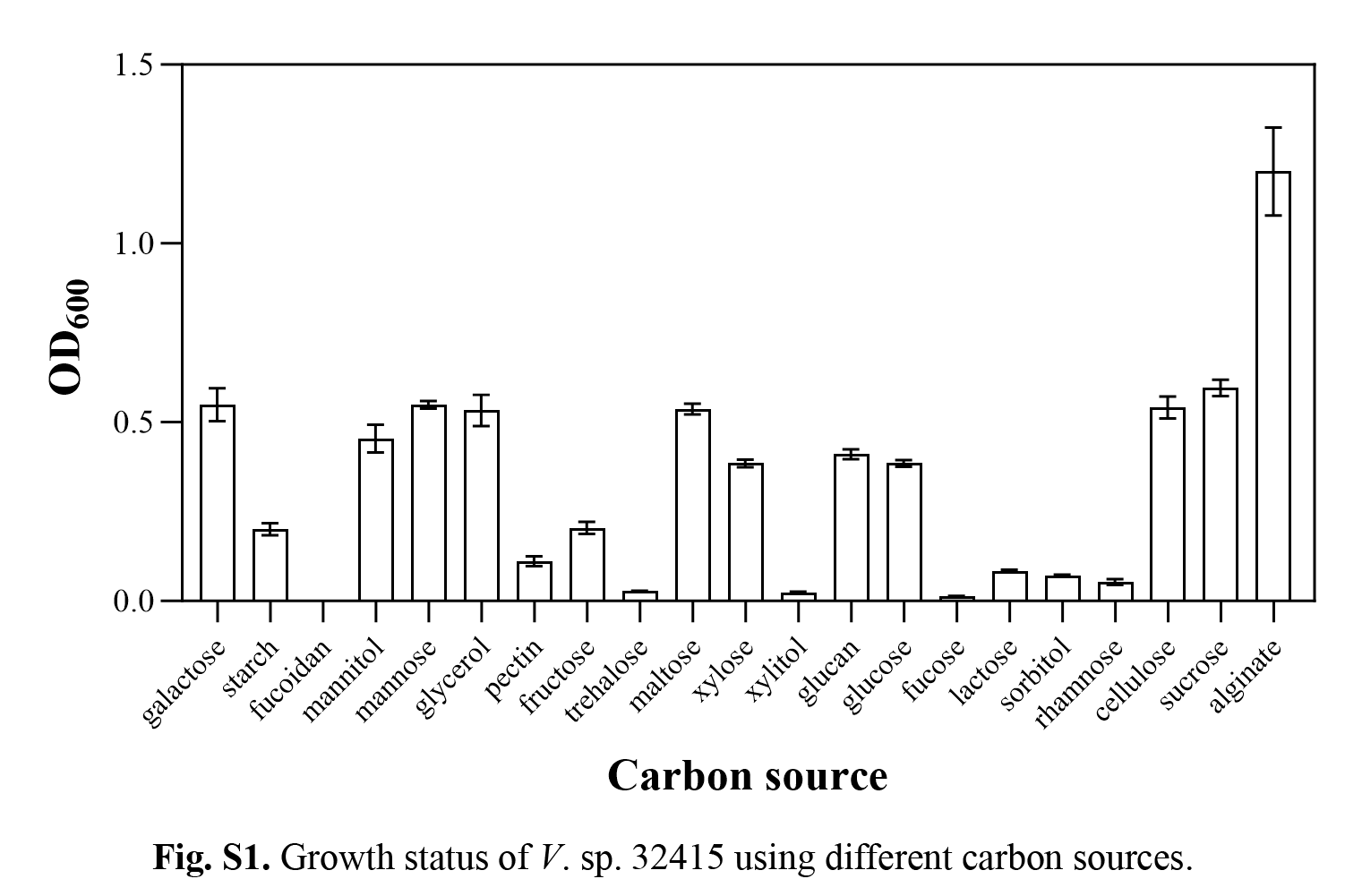

Supplement: Supplementary file 1 [file microorganisms-13-02385-s001.zip › Figure S1.tif]

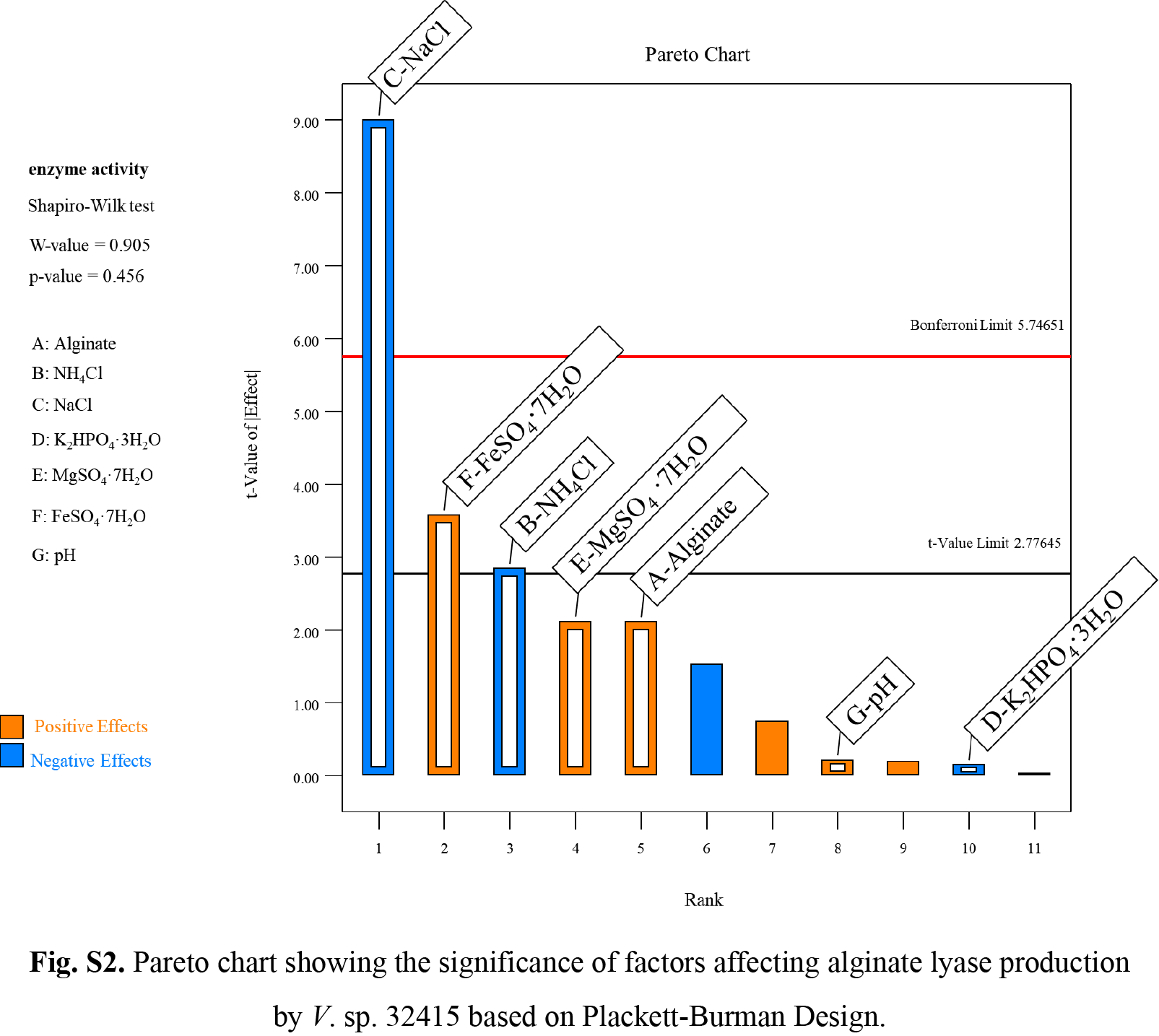

Supplement: Supplementary file 1 [file microorganisms-13-02385-s001.zip › Figure S2.tif]
